# Computational Modeling, High-Level Soluble Expression and In Vitro Cytotoxicity Assessment of Recombinant *Pseudomonas aeruginosa* Azurin: A Promising Anti-Cancer Therapeutic Candidate

**DOI:** 10.3390/pharmaceutics15071825

**Published:** 2023-06-26

**Authors:** Shakira Aslam, Hafiz Muzzammel Rehman, Muhammad Zeeshan Sarwar, Ajaz Ahmad, Nadeem Ahmed, Muhammad Imran Amirzada, Hafiz Muhammad Rehman, Humaira Yasmin, Tariq Nadeem, Hamid Bashir

**Affiliations:** 1 Centre for Applied Molecular Biology, University of the Punjab, Lahore 54590, Pakistan; shakiramfm20@camb.edu.pk (S.A.); rehman.phd20@camb.edu.pk (H.M.R.); 2 School of Biochemistry and Biotechnology, University of the Punjab, Lahore 54590, Pakistan; muzzammel.phd.ibb@pu.edu.pk; 3 Department of Human Genetics and Molecular Biology, University of Health Science, Lahore 54600, Pakistan; 4 South Surgical Ward, King Edward Medical University/Mayo Hospital, Lahore 54000, Pakistan; mzeeshansarwar@kemu.edu.pk; 5 Department of Clinical Pharmacy, College of Pharmacy, King Saud University, Riyadh 11451, Saudi Arabia; ajukash@gmail.com; 6 Centre of Excellence in Molecular Biology, University of the Punjab, Lahore 54000, Pakistan; nadeem.ahmed@cemb.edu.pk; 7 International Center for Genetic Engineering and Biotechnology, Galleria Padriciano, 99, 34149 Trieste, TS, Italy; 8 Department of Pharmacy, COMSATS University Islamabad, Abbottabad Campus, Abbottabad 22010, Pakistan; imranamirzada@cuiatd.edu.pk; 9 School of Pharmaceutical Sciences, Jiangnan University, Wuxi 214082, China; 10 University Institute of Medical Laboratory Technology, Faculty of Allied Health Sciences, The University of Lahore, Lahore 54000, Pakistan; 11 Department of Infectious Diseases, Faculty of Medicine, South Kensington Campus, Imperial College, London W2 1NY, UK; humaira.yasmin@comsats.edu.pk; 12 Department of Biosciences, COMSATS University Islamabad, Islamabad 54000, Pakistan

**Keywords:** azurin, p53, EphB2, cancer therapy, breast cancer, *Pseudomonas aeruginosa*

## Abstract

Azurin is a natural protein produced by *Pseudomonas aeruginosa* that exhibits potential anti-tumor, anti-HIV, and anti-parasitic properties. The current study aimed to investigate the potential of azurin protein against breast cancer using both in silico and in vitro analyses. The amino acid sequence of Azurin was used to predict its secondary and tertiary structures, along with its physicochemical properties, using online software. The resulting structure was validated and confirmed using Ramachandran plots and ERRAT2. The mature azurin protein comprises 128 amino acids, and the top-ranked structure obtained from I-TASSER was shown to have a molecular weight of 14 kDa and a quality factor of 100% by ERRAT2, with 87.4% of residues in the favored region of the Ramachandran plot. Docking and simulation studies of azurin protein were conducted using HDOCK and Desmond servers, respectively. The resulting analysis revealed that Azurin docked against p53 and EphB2 receptors demonstrated maximum binding affinity, indicating its potential to cause apoptosis. The recombinant azurin gene was successfully cloned and expressed in a BL21 (DE3) strain using a pET20b expression vector under the control of the pelB ladder, followed by IPTG induction. The azurin protein was purified to high levels using affinity chromatography, yielding 70 mg/L. In vitro cytotoxicity assay was performed using MCF-7 cells, revealing the significant cytotoxicity of the azurin protein to be 105 µg/mL. These findings highlight the potential of azurin protein as an anticancer drug candidate.

## 1. Introduction

Despite tremendous improvements in cancer diagnosis and treatment, tumors remain one of the world’s top causes of death. One of the most important issues with current therapy is the adverse chemotherapeutic effects and drug resistance against various cancers [1]. Therefore, it is crucial to discover novel anticancer medications and substances. Bacterial proteins and peptides have the potential to be a new family of bioactive compounds and anticancer drugs.

Despite being harmful bacteria, *Pseudomonas aeruginosa* generates some therapeutic compounds, such as azurin. Apart from *Pseudomonas aeruginosa*, other naturally occurring bacteria that produce azurin include *Bordetella bronchiseptica* and *Alcaligenes denitrificans* [2]. *Pseudomonas aeruginosa* produces azurin as a protective periplasmic protein against copper poisoning. Azurin has a higher affinity than other anti-cancerous proteins for reaching the target spot and carrying out its activity. The anti-cancerous action of azurin is probably brought about by the 50–70 (p28) domains, having 28 amino acids [3]. Azurin’s anticancer action has been credited to various components, including the accompanying: (I) cancer development restraint by avoiding angiogenesis by lowering the action of VEGFR-2 tyrosine kinase; (ii) obstruction with protein articulation of P-cadherin and inhibiting tumor growth; (iii) impedance with the Eph-Ephrin Pathway; (iv) p53 protein stabilization and (v) redox homeostasis regulation.

Bioinformatics advancements have enabled the in silico analysis of the designed proteins or peptides before being used for various treatments. Before performing an in vitro assay, this bioinformatics technique facilitates selecting the best possible compounds. Numerous research groups have used bioinformatics to create various in silico compounds [4,5,6]. A bioinformatics approach and computational techniques, such as molecular dynamics (MD) simulations, are used first to analyze new drug candidates in silico.

MD simulation can study intriguing properties such as molecular interactions, physical processes, minimum geometries of proteins, drug-binding free energies, and the precise motion of molecules or atoms over a set time. Additionally, this method has evaluated positioning, wrapping, distance, interactions between residues, and the potential involvement of hazardous and targeted moieties in assessing and designing target proteins [7,8,9]. Firstly, in the current study, using in silico techniques, we predicted the structural physiochemical and stability properties of the *Pseudomonas aeruginosa* azurin protein. Secondly, due to the significance of azurin protein in numerous therapeutic applications, high-yield expression and purification are widely desired. For the production of several recombinant proteins, *E. coli* has always been the preferred organism [10].

Although *E. coli* can express eukaryotic genes at their highest levels in the cytoplasm, this process has several drawbacks, including the inability to create disulfide bridges, inactive proteins, and inclusion bodies. The issue of misfolded and biologically inactive proteins can be resolved by directing the target protein to the periplasmic region of the bacterial host [11].

Recombinant proteins expressed in the periplasm rather than the cytoplasm have several major advantages, including a reduction in proteolytic degradation and a more suitable environment for producing disulfide bonds and protein folding [12]. Secondly, the periplasmic area has significantly less proteolytic activity than the extracellular space, creating a favorable environment for expressing smaller polypeptides. Periplasmic space expression has an advantage over cytoplasmic expression in that substrates are easily accessible in the periplasm due to their inability to penetrate the inner cytoplasmic membrane [13].

The main focus of the current study was the in-frame cloning of the recombinant azurin gene in a pET-20 b (+) vector with pelB-leader sequence at the N-terminal of cloned gene and to obtain periplasmic expression in the BL21DE3 *E. coli* strain. The expressed protein was purified via a single-step affinity chromatography purification process, and the cytotoxicity was evaluated against the MCF-7.

## 2. Materials and Methods

### 2.1. Protein Selection and Primary Structure Analysis

The *Pseudomonas aeruginosa* azurin gene sequence was retrieved from NCBI with accession number M30389. The amino acid sequence from Uniprot (Uniprot ID: B3EWN9) was used further to evaluate the azurin protein’s properties. Quantitative determination of azurin protein purity was performed via solubility analysis. The analysis of the physiochemical properties and solubility was performed via the ExPASy Protparam tool (https://web.expasy.org/protparam/protparam-doc.html, accessed on 1 September 2022).

### 2.2. Secondary and Tertiary Structure Prediction

The secondary structure of azurin protein was predicted using the PsiPred tool, which uses the PSI-blast method to explore the secondary structure. However, I-TASSER (Iterative Threading ASSEmbly Refinement) was used to predict the tertiary structure of azurin protein and the most authentic structure with the highest C-score.

### 2.3. Validation and Refinement of Azurin Protein Tertiary Structure

The validation of the quality of the 3D structure was conducted using the SAVES 6.0 server. Statistics of non-bonded interactions among various atom types in azurin protein were analyzed through the application of the ERRAT value. The stereo-chemical quality of the azurin protein structure was validated by analyzing the residue-by-residue geometry via PROCHECK. The GalaxyRefine web server is considered one of the most authentic tools for refining tertiary structures. In its refinement method, side chain rebuilding and repacking were initially performed. In this way, the tertiary structure of the azurin protein was validated and refined through the GalaxyRefine server.

### 2.4. Antigenicity and Allergenicity of Azurin Protein

Azurin protein was analyzed using VaxiJen 2.0 and AllerTOP online software to predict antigenicity and allergenicity.

### 2.5. Structure Refinement of Receptor Molecule for Docking

Two receptor molecules (p53 and Eph-B2) were selected to determine the interaction of azurin protein with them. The p53 transcription activation domain and Eph-B2 receptor structure were retrieved from the RCSB Protein Data Bank with PDB ID: 8F2H and 1SHW, respectively. The structures were purified, and the missing residues were built by the Pymol tool before proceeding to dock.

### 2.6. Molecular Docking Azurin-p53 and Azurin-EphB2 Complexes

The present study used the HDOCK server for docking between the selected receptors (p53 and EphB2) and azurin protein. Azurin binds with EphB2 and p53, resulting in anti-cancerous activity through the inhibition of tumor growth and the induction of cancer cell apoptosis. HDOCK is an online web server available at http://hdock.phys.hust.edu.cn/, accessed on 21 September 2022. The HDOCK server automatically predicts an interaction between proteins via the hybrid algorithm of template-based and template-free docking. Based on the docking and confidence score, the best-docked complex was selected for further analysis.

### 2.7. Molecular Dynamics Simulation

The Desmond module of Schrodinger was exploited to conduct the MD simulation studies. The dynamic behavior and stability of the protein–protein complexes were investigated using their docked poses. The protein–protein complex was preprocessed using the Protein Preparation Wizard of Maestro, which included complex optimization and minimization. All of the systems were prepared using the System Builder tool. The solvation of the complexes was performed with the simple point-charge (SPC) water model with an orthorhombic box, along with a 10-Å distance from the edge of the box, and the system was neutralized with Na^+^/Cl^−^ ions. To mimic the physiological conditions, 0.15 M sodium chloride (NaCl) was added. The potential energy of the protein complex was minimized by employing the NPT ensemble. The molecular dynamics simulations were performed at 300 K temperature and 1 atm pressure for 100 ns, and NPT production ran under the OPLS4 force field. The models were relaxed before the simulation. The short-range electrostatic interactions were calculated using the particle mesh Ewald method [14]. The cutoff radius in the Coulomb interactions was 9.0 Å. The water molecules were explicitly described using the simple point charge model [15]. The Martyna–Tuckerman–Klein chain coupling scheme [16] with a coupling constant of 2.0 ps was used for the pressure control, and the Nosé–Hoover chain coupling scheme [16] was used for the temperature control. The trajectories were saved for examination after every 100 ps, and the simulation’s stability was verified by comparing the root mean square deviation (RMSD) of the protein complex over time. The projected changes in their conformation from the initial structure over the entire simulation period were expressed as root mean square deviation (RMSD) and root mean square fluctuation (RMSF) for MD simulations. A detailed description of the methodology can also be found elsewhere [17,18,19,20].

### 2.8. Cloning and Expression of Recombinant Azurin Gene

To express the protein, the synthesized gene of azurin in pUC 57 from the genescript was subcloned into the pET20b vector under the T7 promoter. Cloning was confirmed via PCR amplification and restriction digestion with specific *Nco1* and *Xho1* restriction enzymes and transformed into BL21 (DE3) *E. coli* strains with the CaCl_2_ method. The eight to ten colonies were grown on the LB medium with appropriate antibiotics at 37 °C for 24 h to select the positive clones, followed by induction with a final concentration of 1 mM IPTG. SDS–PAGE was used to evaluate the expression level of the positive clones.

### 2.9. Shake Flask Fermentation of Recombinant Azurin

Fermentation was performed to express the recombinant azurin protein in high-level shake flasks. The positive transforming was grown in LB media supplemented with specific antibiotics at 37 °C for 24 h. The overnight grown culture was further inoculated into 1 L M9 medium, adding the suitable antibiotics and other salts in a shaking incubator at 200 rpm at 37 °C. The grown culture with continuous shaking in the incubator at 37 °C was then induced with IPTG to maintain the final concentration of 1 mM. After an 18 h induction period, the cell culture was subjected to centrifugation, and the collected biomass was stored at −20 °C for further use.

### 2.10. Preparation of Periplasmic Fraction

To prepare the preplasmic fraction, the osmotic shock method described by Ausubel et al. [21] was used. The shake flask fermentation biomass was resuspended in a buffer of 30 mM Tris-HCl with pH 8.0, 20% sucrose, and 0.5 M EDTA, followed by centrifugation at 10,000 rpm for 10 min. Then, the pellet was resuspended in 5 mM MgSO_4_, and the solution was centrifuged again for 10 min at 10,000 rpm to collect the supernatant.

### 2.11. Recombinant Azurin Protein Purification

#### 2.11.1. Diafiltration of Periplasmic Fraction

Prior to purification to remove the salts and other impurities, the periplasmic fraction of the azurin protein was buffer exchanged with 20 mM of sodium phosphate buffer (pH 7.4) and concentrated with Tangential flow filtration systems, passing the solution through an Opticap filter (MilliporeSigma, Burlington, NY, USA) and nitrocellulose membrane cartridge (0.45 μm). A peristaltic pump was used to pass the sample solution and buffer at a 3 mL/min rate through a nitrocellulose membrane cartridge (0.45 μm). After buffer exchange, the sample solution was concentrated to 20% of the total volume and further used to precede the purification process.

#### 2.11.2. Purification

The periplasmic fraction was purified by loading the desalted protein onto a nickel Sepharose-packed column pre-equilibrated with buffer A, ((20 mM sodium phosphate, pH 7.4, 0.5 M sodium chloride, and 0.01% Tween 20). The protein-loaded column was washed with buffers A and B (20 mm sodium phosphate pH7.4, 0.5 M sodium chloride, and 0.01 M imidazole) to remove the loosely bound protein and impurities. Finally, the target protein was eluted with elution buffer, and the fractions were pooled after analysis on the SDS-PAGE.

### 2.12. Characterization of Purified Azurin Protein

Protein purity was confirmed by running purified azurin protein samples on 12% SDS-PAGE for 90 min at 110 volts. After running the gel, coomassie brilliant blue stain was used to stain the gel, followed by destaining with destain solution for clear bands visualization. The purified protein bands that underwent Western blotting were transferred to a nitrocellulose membrane, and 5% skimmed milk in PBST was used to block the nitrocellulose membrane. After blocking, membrane washing was conducted with PBST and the membrane was treated with primary His-tag antibody followed by 2 h of incubation. The membrane was then again washed with PBST and treated with secondary His-tag alkaline phosphatase-conjugated antibody followed by 1 h of incubation. The membrane was then treated with NBT/BCIP after washing it with autoclaved water to visualize the protein bands.

### 2.13. Bioassay of the Recombinant Azurin Protein

To perform the activity assay of the purified recombinant azurin protein, the MCF-7 and MCF-10F cells were cultured in RPMI containing 10% FBS and antibiotics (penicillin and streptomycin) in a 5% carbon dioxide incubator at 37 °C. An MTT cell proliferation assay was used to determine purified azurin protein cytotoxicity against MCF-7 and MCF-10F cells. The cells were cultivated for 72 h to reach the desired cell densities and then treated with different concentrations of azurin (10–140 M) in triplicate, with MCF-7 and MCF-10F cells serving as a control. A protein-treated 96-well plate was incubated at 37 °C for twenty-four hours in a CO_2_ incubator. Then, 10 µL of MTT reagent was added to each well, followed by a four-hour incubation. After incubation, the well content was replaced with 100 µL DMSO to dissolve formazan crystals. The absorbance of the plate was read at 570 nm using an ELISA plate reader. Using the below-mentioned formula, cell viability was calculated.

(1) $$\mathrm{Percentage}\;\mathrm{cell}\;\mathrm{viability}=\frac{\left(\mathrm{test}\;\mathrm{wavelength}\;570\;\mathrm{nm}-\mathrm{reference}\;\mathrm{wavelength}\;630\;\mathrm{nm}\right)\times 100}{\mathrm{Control}\;570\;\mathrm{nm}-630\;\mathrm{nm}\;}$$

## 3. Results

### 3.1. Primary Structure Analysis of the Azurin Protein

The primary structure of azurin protein contains 148 amino acids. A signal peptide of 20 amino acids was removed before analyzing the physiochemical and structural parameters of azurin. The primary structure reveals a variety of physiochemical properties of the protein. A protein’s ability to depict a therapeutic effect is related to the influence of its various physicochemical properties on the biomolecule that it interacts with. ProtParam computes several physiochemical properties that could infer from protein sequence. ProtParam calculates theoretical pI, molecular weight, amino acid content, instability index, estimated half-life, aliphatic index, and the grand average of hydropathicity (GRAVY) (Table 1).

According to the results, the azurin protein is acidic, having 15 negatively charged residues. The molecular weight and theoretical isoelectric point of the azurin protein were predicted as being 13.9 kDa and 5.72, respectively. The azurin protein instability index was evaluated as being 16.74, which assures the stability of azurin protein. The azurin protein aliphatic index was calculated to be 70.78. The estimated half-life of azurin protein is calculated to be 4.4 h in mammalian cells, >20 h in yeast, and >20 h in *E. coli*.

### 3.2. Azurin Secondary and Tertiary Structure Analysis

**Figure 1:**
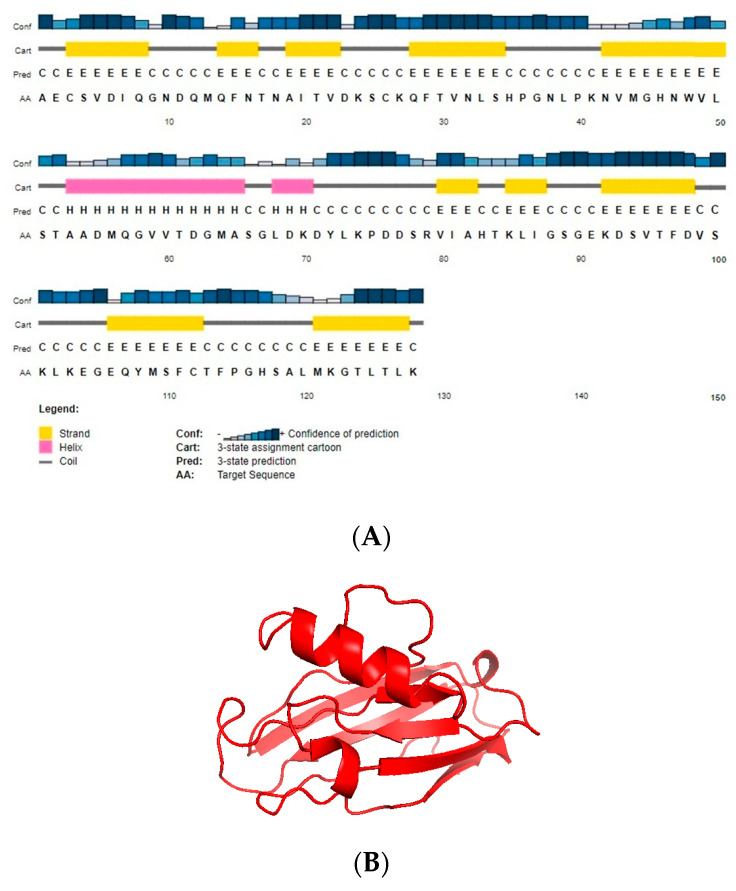
(**A**) Secondary structure of azurin protein predicted using the PsiPred tool showing alpha helix residues (pink), extended strand residues (yellow), and random coil residue (black) the content in azurin protein. (**B**) The tertiary structure of azurin protein predicted via the I-TASSER tool.

Protein function directly relies on interactions with other proteins, structures, and locations within cells, tissues, and organs. The structure and function of protein enable the identification of protein biomarkers associated with specific disease states and provide potential therapeutic targets. The protein sequence of azurin (Uniprot ID: B3EWN9) was used to predict the secondary structure via the Psipred tool (Figure 1A), while the 3D structure was predicted through the use of the I-TASSER tool (Figure 1B). A template with PDB ID: 4azu was used by an I-TASSER server for tertiary structure prediction. The target protein and the template protein shared 100% identity, with a GMQE score of 0.97.

**Figure 2:**
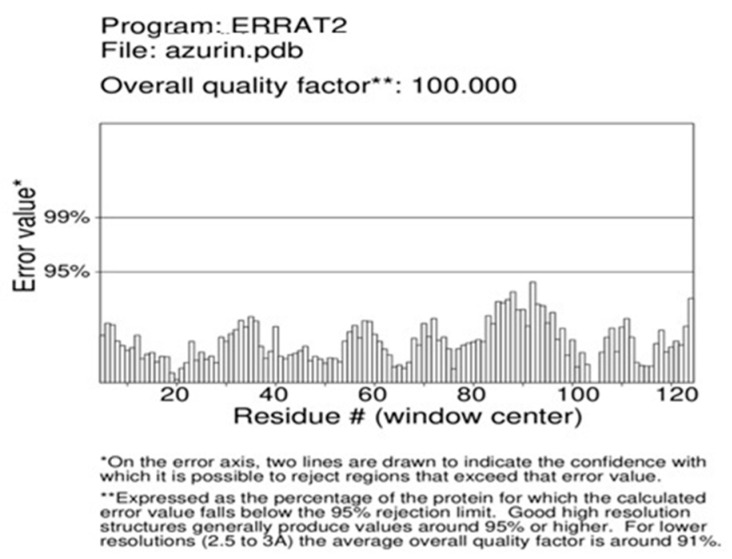
ERRAT value index of GalaxyRefined structure of azurin protein having a quality factor of 100%.

**Figure 3:**
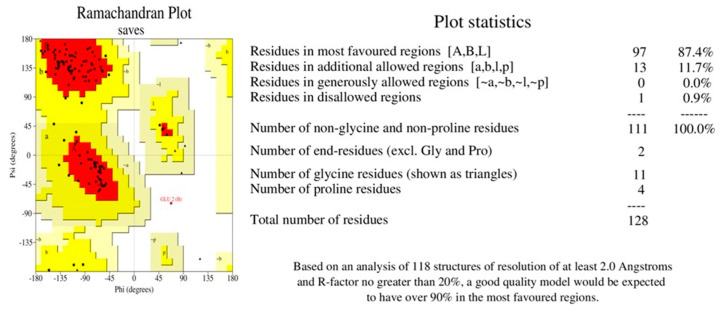
Ramachandran plot of GalaxyRefined structure of azurin protein showing residues (black dots) in the favored (Red), allowed (Yellow), and disallowed (White) regions.

The azurin protein PDB structure was submitted to Procheck to evaluate the modeled protein stereochemistry. Ramachandran plot analysis showed that 79.5% of the residues lie in the most favored region. The refinement of the structure was performed using the GalaxyRefine tool. Among the five models given by the GalaxyRefine tool, one model was selected after validation for SAVES analysis. The final model, after validation, was selected based on the greater ERRAT value and Ramachandran-favored residues. The ERRAT value of the final model was 100% (Figure 2), with 87.4% residues in Ramachandran’s most favored region (Figure 3).

### 3.3. Prediction of the Allergenicity and Antigenicity of Azurin Protein

To use a protein in therapeutics, it is essential to declare it safe by determining its antigenicity and allergenicity. To predict azurin protein allergenicity, the protein amino acid sequence was imported to AllerTop software and it was deduced that the protein was nonallergen. The protein antigenic nature was predicted using the VaxiJen server based on its physical and chemical properties. To differentiate antigenic and non-antigenic proteins, the threshold was set to 0.4. Azurin protein was predicted to be antigenic, having a score of 0.49.

### 3.4. Molecular Docking of Azurin with p53 and EphB2

Numerous biological functions, including cell-to-cell communication, metabolic regulation, and control of development, are handled by protein–protein interactions [22]. To estimate the interaction between azurin protein and the selected receptors, protein–protein docking was performed via the HDOCK server. The docking server gave multiple models, and after analyzing them individually, one model of each EphB2–azurin and p53–azurin docked complex with the lowest docked energy was selected. The docking scores and confidence score of the p53–azurin docked complex was −266.70 and 0.9117, and the EphB2–azurin docked complex was −198.82 and 0.7264, respectively. These docked complexes were selected for interaction and simulation studies.

### 3.5. Molecular Dynamics Simulation

**Figure 4:**
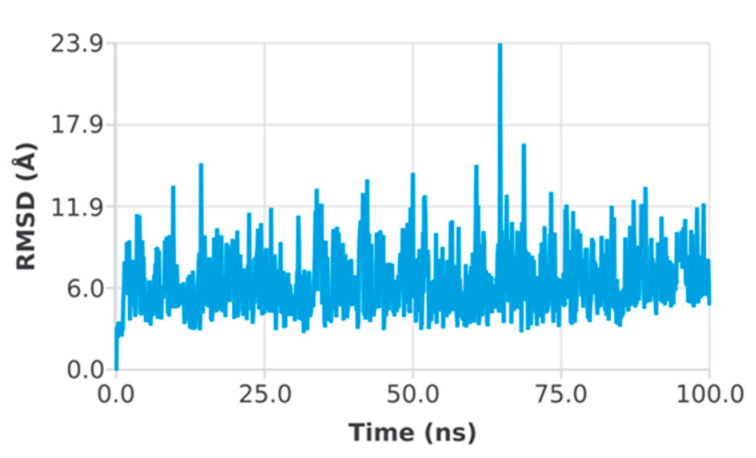
Root mean square deviation plot of EphB2–azurin complex.

**Figure 5:**
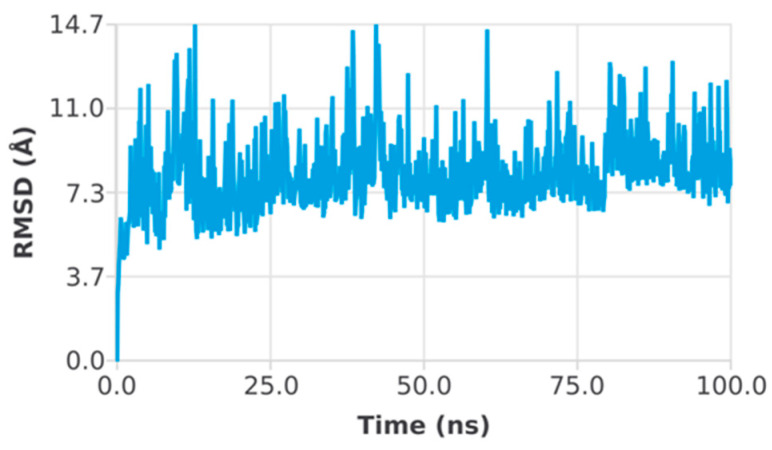
Root mean square deviation plot of p53–azurin complex.

High binding energy top hits were subjected to molecular dynamics simulations. The predicted conformational changes from the original structure across the simulation period were shown as root mean square deviation (RMSD). Moreover, root mean square fluctuation (RMSF) measurements were used to indicate structural stability, atomic mobility, and residue flexibility during the interaction of a protein with a hit. The peaks of the RMSF graph represent the protein’s fluctuation component during the simulation. More modifications occur in the N- and C-terminal regions of the protein than in the other regions. Alpha helices and beta strands exhibit less volatility since they are stiffer than the loop component of the protein’s unstructured region. The RMSD of the EphB2–azurin complex showed a small deviation at almost 60 ns, and then the system converged throughout the simulation. It indicates the protein–protein complex’s stability and whether the simulation has equilibrated (Figure 4). The RMSD of the p53–azurin complex showed a deviation at 10 ns and then a small deviation at almost 77 ns. After that, the system was equilibrated, and the simulation converged (Figure 5).

**Figure 6:**
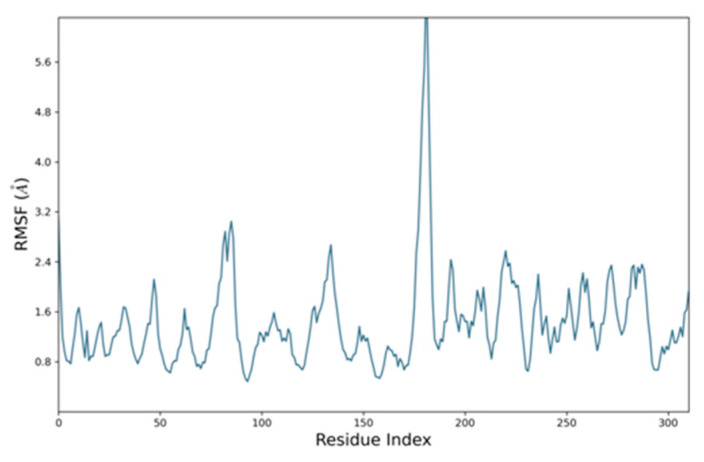
Root mean square fluctuation plot of EphB2–azurin complex.

**Figure 7:**
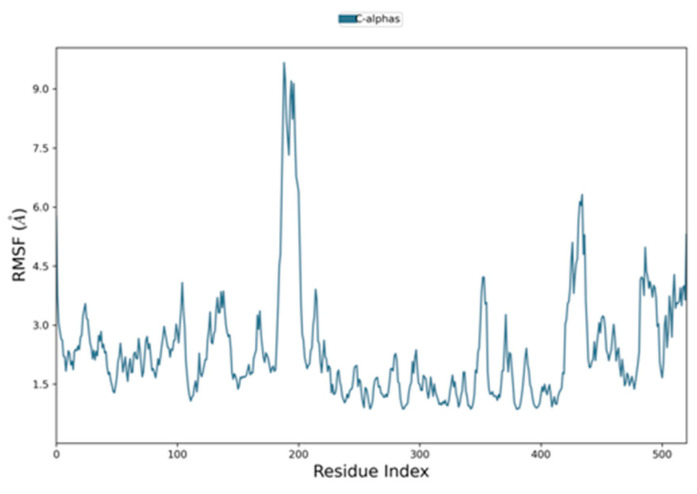
Root mean square fluctuation plot of p53–azurin complex.

Similarly, root mean square fluctuation (RMSF) is useful for characterizing local changes along the protein chain. For the RMSF of the EphB2–azurin complex, the fluctuations were in the range of 3 Å except where both proteins started interacting with each other (Figure 6). Similarly, the RMSF of the p53–azurin complex showed fluctuation from ASP 61 to PRO 72, and the remaining part fluctuated in range, indicating stability (Figure 7).

### 3.6. Protein–Protein Interactions of Azurin Protein with p53 and EphB2 Receptors

**Figure 8:**
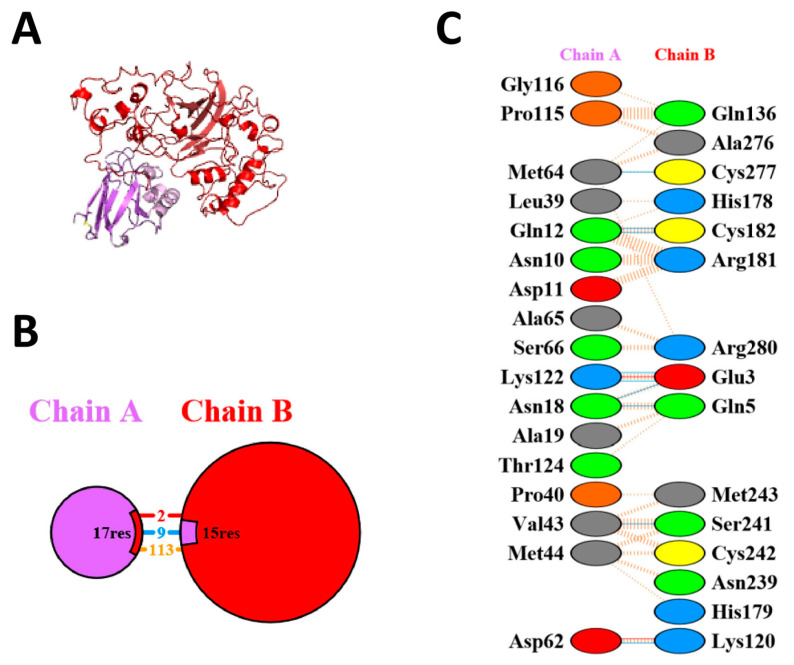
The protein–protein interactions analysis of p53–azurin docked complex. (**A**) The full-docked complex of p53–azurin. The purple region shows azurin, and the red region shows p53. (**B**) Chain A shows azurin, and chain B shows p53. The blue line shows hydrogen bonds, the red shows salt bridges, and the orange represents non-bonded interactions. (**C**) The detailed view of types of interactions among the residues.

**Figure 9:**
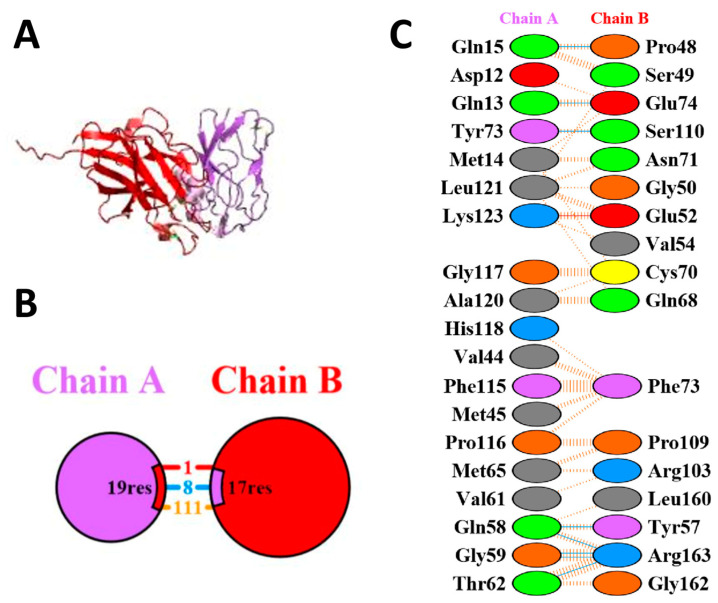
The protein–protein interactions analysis of the EphB2–azurin docked complex. (**A**) The full docked complex of EphB2–azurin. The purple region shows azurin, and the red region shows EphB2. (**B**) Chain A shows azurin, and chain B shows EphB2. The blue line shows hydrogen bonds, the red shows salt bridges, and the orange represents non-bonded interactions. (**C**) The detailed view of types of interactions among the residues.

PDBSum analyzed the protein–protein interactions of the p53–azurin and EphB2–azurin complexes, and then the interactions were analyzed. The number of hydrogen bonds, non-bonded interactions, and salt bridges were analyzed among both docked complexes. There are nine hydrogen bonds, two salt bridges, and 113 non-bonded interactions between azurin and p53 complex (Figure 8). There are eight hydrogen bonds, one salt bridge, and 111 non-bonded interactions between the azurin and the EphB2 complex (Figure 9).

### 3.7. Cloning and Expression of Azurin Protein

**Figure 10:**
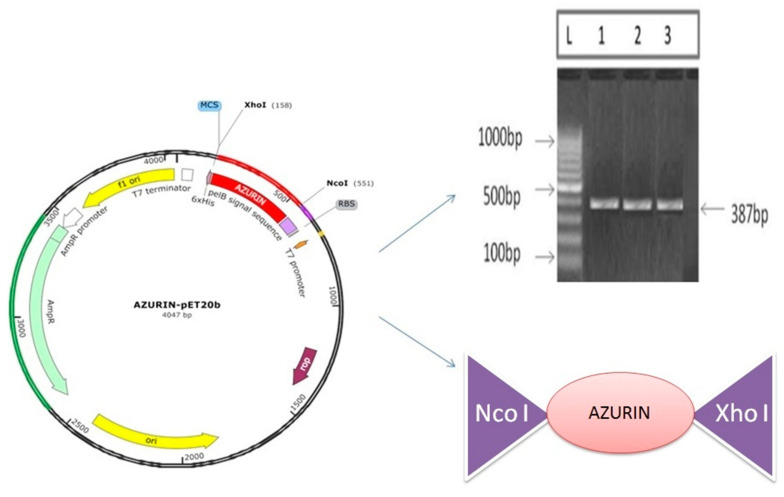
Amplification and cloning of the recombinant azurin into pET20b expression vector.

The synthesized gene of recombinant azurin was cloned into the pET20b expression vector at Nco1 and Xho1 sites without the stop codon to utilize the His-Tag and was designated as azurin–pET20b. The expression cassette of recombinant azurin–pET20b was transformed in the BL21 (DE3) strain of *E. coli* to study the expression (Figure 10).

### 3.8. Expression and Purification of Recombinant Azurin Protein

**Figure 11:**
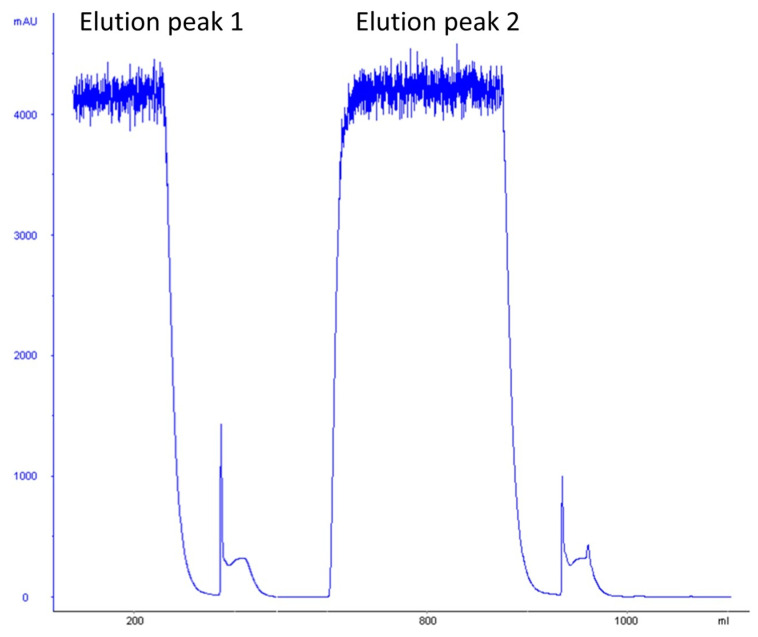
Chromatogram of azurin protein purification via affinity chromatography.

Shake flask fermentation was performed to express azurin protein on a large scale. The periplasmic fraction of the IL shake flask biomass was used for further purification via affinity chromatography. The protein was eluted with 1 M imidazole, and pure fractions were pooled for further study. Figure 11 represents the purification via affinity chromatography, while Table 2 represents the overall summary of the total yield of the recombinant azurin protein obtained from the IL batch culture via single-step affinity chromatography.

### 3.9. Characterization of Purified Azurin Protein

**Figure 12:**
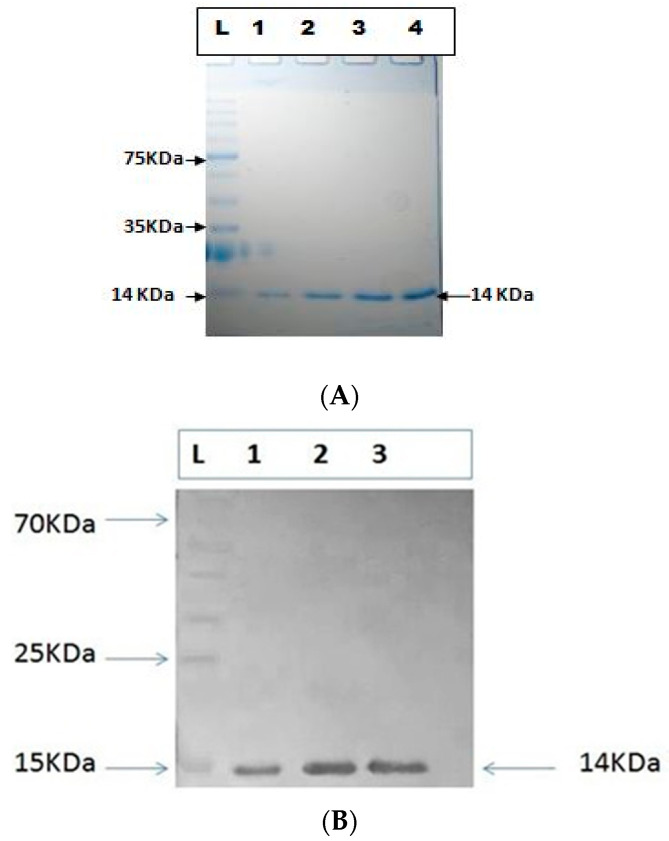
(**A**) SDS-PAGE analysis of the purified azurin protein. (**B**) Western blotting of purified azurin protein.

To check the purity of the expressed protein, purified recombinant azurin protein was analyzed via SDS-PAGE, and a single band with a size of 14 kDa was observed to represent greater than 98% purity of the protein. Furthermore, Western blot also confirmed the purity of the recombinant azurin protein (Figure 12A,B).

### 3.10. Azurin Protein Biological Activity Assay

**Figure 13:**
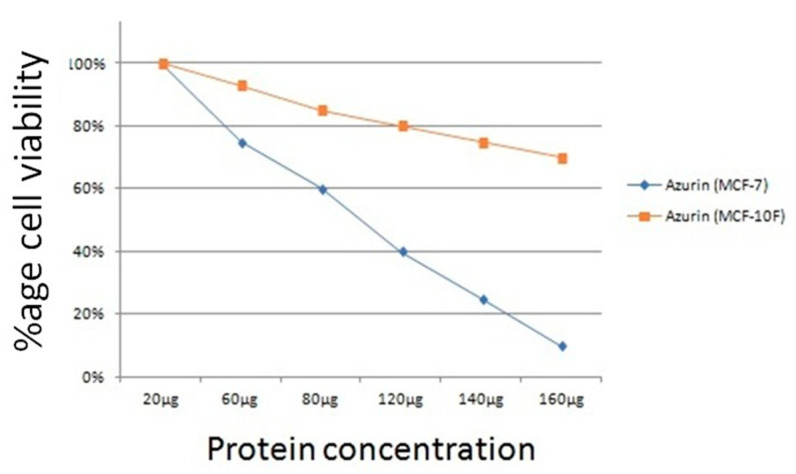
Cytotoxicity of azurin protein against MCF-7 cell lines.

The cytotoxicity of azurin was evaluated against MCF-7 and MCF-10F cells. The cells were treated for 24 h with different azurin (20–160 µg/mL) concentrations. After measuring cell viability via the MTT assay, it was observed that azurin repressed MCF-7 cell viability in a dose-dependent manner (azurin, 20 µg/mL: 98.85 ± 5.107, azurin, 60 µg/mL: 73.95 ± 3.387, azurin, 80 µg/mL: 59.13 ± 3.136, azurin, 120 µg/mL: 40.26 ± 1.108, azurin, 140 µg/mL: 26.09 ± 2.492, azurin, 160 µg/mL: 9.95 ± 0.854). Figure 13 shows the azurin protein cytotoxic effect after 24 h, and the results showed a major decrease in MCF-7 cell survival with azurin, with an IC_50_ of 105 μg/mL. Moreover, the apoptotic effect of azurin was significantly less in normal cell line MCF-10F (azurin, 20 µg/mL: 99.26 ± 6.71, azurin, 60 µg/mL: 93.57 ± 7.026, azurin, 80 µg/mL: 85.09 ± 2.924, azurin, 120 µg/mL: 80.41 ± 1.969, azurin, 140 µg/mL: 74.87 ± 4.347, azurin, 160 µg/mL: 68.57 ± 2.902).

## 4. Discussion

Chemotherapy and radiotherapy are conventional treatment strategies to treat cancer, but due to their developed resistance in cancer cells, new treatment strategies need to be developed to combat cancer. Recently, bacterial proteins and peptides have been considered potent candidates to target and destroy cancer cells. *Pseudomonas aeruginosa* produces a variety of medically essential agents, such as antibiotics, toxins, and proteins. Azurin is also a defensive protein produced by *Pseudomonas aeruginosa* [23]. Azurin is involved in various signaling pathways, but two segments have been discovered to interact with signaling targets. One is the p28 segment actively interacting with p53 [24], and the other is the C-terminal segment involved in interaction with EphB2 [25]. P53 is a tumor suppressor protein with 393 amino acid residues. There are three domains of p53: tetramerization domain, transcription activation domain, and DNA binding domain [26]. When *Pseudomonas aeruginosa* azurin protein enters the cells, it reacts with p53 and forms a stable complex enhancing the level of p53 in cells. When the p53 level enhances, the cell undergoes apoptosis due to increased Bax formation [23]. Ephrin and its receptors are involved in tumor up-regulation [27,28,29]. When Ephrin binds with its receptors, a hetero-tetramer activates the tyrosine kinase domain of Ephrin receptors. In addition to the p28 domain (amino acid residue 50–77), azurin also contains another domain (C-terminal) that facilitates the binding of azurin with EphB2 receptors, resulting in anti-cancerous activity and inhibiting the growth of tumors. Azurin shares structural homology with ephrinB2, a ligand of the EphB2 tyrosine kinase receptor. When binding with the Ephrin-B2 receptor, azurin inhibits the autophosphorylation of tyrosine kinase residues, ultimately inhibiting cancer cells [25].

The use of bioinformatics approaches results in a significant reduction in cost, time, and experimental failure attempts. The prediction, analysis, and interpretation of clinical and preclinical findings are all greatly aided by bioinformatics technologies [30]. However, the predicted findings through bioinformatics techniques are not always concordant with the experimental results. With the advancement in bioinformatics techniques and software regarding the relationship between protein structure and functions, these techniques could play an important role in developing a suitable protein as a targeted drug. Considering the therapeutic potential and its involvement in various signaling pathways before expressing the recombinant azurin protein, we performed in silico analysis. The bioinformatics analysis revealed that the nature of azurin protein is basic, with a molecular weight of 14 kDa and pI value of 5.72, and azurin is a nonallergen. Azurin was found to be antigenic for tumor cells, with a value of 0.49. According to aliphatic index calculations, the azurin protein was predicted to be a stable protein. Sequence and structural homology serve as the main foundation for protein structure prediction. Because a protein’s function primarily depends on its 3D structure, protein structure prediction or modeling is crucial [31]. Psipred and I-TASSER, respectively, predicted the secondary and tertiary structures of azurin protein. Azurin protein contains 12.5% residues in alpha helix, 43.75% in beta sheets, and 43.75% coils, respectively. The tertiary structure was refined using the GalaxyRefine tool and validated through the use of Ramachandran plot analysis, which shows 87.4% residues in the favored region. Protein–protein interactions are essential for proteins to operate and be regulated during the cell cycle, DNA replication, and cellular signaling. As a result, it is crucial to identify protein interactions while developing new drugs [32]. Molecular docking approaches aim to predict a ligand–protein and protein-optimal protein’s binding mode. Docking analysis shows a single snapshot of complex physiological movement. Therefore, dynamic simulation studies are required to study the interaction among proteins in a flexible environment [33]. A computational method called molecular dynamics (MDs) models the dynamic behavior of molecular systems as a function of time [34]. Simulation studies show that the protein is stable in a complex form with respective receptors, and less deformation is exhibited by its residues during complex formation.

Soluble protein production facilitates the recombinant protein isolation from the *E. coli* periplasmic fraction rather than a whole-cell lysate [35,36]. The azurin gene was cloned in the pET20b expression vector and expressed in the BL21 (DE3) strain under the T7 promoter. Numerous purification processes have been developed to efficiently purify recombinant proteins using an *E. coli* expression system, including multistep HPLC and FPLC chromatography and ion exchange purification, which is not undesirable for large-scale purification due to their multistep process and low product yield. Affinity chromatography is a powerful, simple technique for separating biological macromolecules. This method emphasizes excellent resolution, selectivity, and capacity in most protein purification methods [37]. In the recombinant technology, expression facilitated protein labeling with His-tag, and the purification and downstream processing became easy [36]. Recombinant azurin protein was purified with affinity chromatography with a yield of 70 mg/L with purity of >98%, characterized by SDS-PAGE and Western blot. The purified protein cell viability assay was performed against the MCF-7 cells to assess the activity. Zaborina et al. reported azurin as being an innovative and interesting anticancer drug with cytotoxic effects against the murine macrophage J774 cell line [38]. Vasu et al. conducted a study to evaluate the cytotoxicity of azurin protein against the MCF-7 cell line and concluded that >50 percent of cells were undergone apoptosis [39]. In the current study, we evaluated the cytotoxicity of azurin protein against the MCF-7 cells and observed significant cytotoxicity of azurin protein at 105 µg/mL.

## 5. Conclusions

In the current study, bioinformatics analysis of azurin protein shows that it can be used as an anticancer agent with stable properties. When it makes a complex with p53 and EphB2, it results in the inhibition of cancer progression. Cloning of the azurin gene was successfully performed in the pET20B expression vector, and high-level expression and purification with a yield of 70 mg/L were observed in *Escherichia coli* strain BL21 (DE3). The significant activity of the azurin protein against the MCF-7 cells and low cytotoxic response against normal breast epithelial cell line MCF-10F highlighted the potential candidate as an anticancer drug.

## Figures and Tables

**Table 1:** Physiochemical parameters of azurin computed by the ProtParam tool.

Physiochemical Properties	Theoretical Values
Molecular weight	13.9 kDa
pI	5.72
Number of positively charged residues	12
Number of negatively charged residues	15
Instability index	16.74
Aliphatic index	70.78
GRAVY	−0.322

**Table 2:** Summary of chromatography.

Steps	Total Protein (mg/L)	Azurin Protein (mg/L)	Purity (%)	Step Yield (%)
Periplasmic fraction	200 (±1)	150 (±1)	35 (±1)	96 (±1)
Diafiltration	135 (±1)	107 (±1)	50 (±1)	85 (±1)
IMAC purification	95 (±1)	70 (±1)	>95 (±1)	82 (±1)

## Data Availability

The data presented in this study are available on request from the corresponding author.
